# Distinct regulation of dengue virus-induced inflammasome activation in human macrophage subsets

**DOI:** 10.1186/1423-0127-20-36

**Published:** 2013-06-07

**Authors:** Ming-Fang Wu, Szu-Ting Chen, Shie-Liang Hsieh

**Affiliations:** 1 Institute of Clinical Medicine & Infection and Immunity Center, National Yang-Ming University, Taipei, 112, Taiwan; 2 Genomics Research Center, Academia Sinica, Taipei, 115, Taiwan; 3 Immunology Center, Taipei Veterans General Hospital, Taipei, 112, Taiwan

**Keywords:** Dengue Virus (DV), Inflammasomes, Macrophages (Mϕ), Resting macrophages (M- Mϕ), Inflammatory macrophages (GM- Mϕ), C-type lectin receptor (CLR), CLEC5A

## Abstract

Macrophages (Mϕ) are the major source of inflammatory cytokines and are target cells for dengue virus (DV) replication. However, Mϕ are heterogeneous and their phenotypic and functional diversities are influenced by cytokines that regulate their differentiation, tissue distribution, and defense against invading pathogens. *In vitro*, human primary macrophages are derived from peripheral blood CD14^+^ monocytes in the presence of macrophage colony-stimulating factor (M-CSF) or granulocyte macrophage colony-stimulating factor (GM-CSF). These are essential for developing tissue/resting macrophages (M-Mϕ) and inflammatory macrophages (GM-Mϕ), respectively. While IFN production is similar between M-Mϕ and GM-Mϕ, M-Mϕ cannot produce IL-1β after DV infection. In contrast, GM-Mϕ is more susceptible to DV infection and DV triggers CLEC5A in GM-Mϕ to activate NLRP3 inflammasomes, which in turn release IL-18 and IL-1β that are critical for Th17 activation and contribute to disease severity. Thus, GM-Mϕ is more representative than M-Mϕ for investigating inflammasome activation in dengue infection, and is invaluable for revealing the molecular mechanism of pathogen-induced inflammatory reaction. Distinct phenotypes of macrophage subsets under the influence of M-CSF and GM-CSF raise the question of optimal conditions for culturing primary macrophages to study host-pathogen interaction.

## Review

### Introduction

Dengue virus (DV) is a positive-sense, single-stranded RNA virus that belongs to the *flavivirus* genus of the Flaviviridae family. It is transmitted among humans by the *Aedes* mosquitoes and is prevalent in over 100 tropical and sub-tropical countries, with about 2.5 billion people at risk [1]. Infection causes a spectrum of illness ranging from sub-clinical and mild febrile illness to classical dengue fever (DF) to severe and sometimes fatal hemorrhagic disease [1]. Classical DF is an acute febrile illness that usually occurs in older children and adults and is often characterized by fever, frontal headache, myalgia, arthralgia, nausea, vomiting, and rash lasting 3–7 days [2]. While DF is self-limiting in most cases, it can progress into dengue hemorrhagic fever (DHF) or dengue shock syndrome (DSS). The signaling pathway leading to dengue infection had been unclear until the myeloid Syk-coupled C-type lectin CLEC5A was identified as the therapeutic target of DF/DHF [3].

Macrophages (Mϕ) are thought to originate from hematopoietic stem cells (HSCs) during development and reside in various tissues such as Kupffer cells in the liver, microglia in the brain, alveolar macrophage in the lungs, osteoclast in the bone, and in lymph nodes and other tissues. Tissue macrophages play a broad role in maintaining tissue homeostasis via clearance of senescent cells and tissue remodeling and repair. While macrophage colony-stimulating factor (M-CSF) has been applied to induce monocyte differentiation into macrophages for host-pathogen interaction, recent studies reveal that granulocyte macrophage colony-stimulating factor (GM-CSF) is influential in skewing macrophage differentiation into distinct phenotypes. Hamilton [4] proposed that a constant M-CSF level is necessary to keep the Mϕ population in a resting and homeostatic situation (M-Mϕ or resting macrophage), while local GM-CSF elevation during infection triggers Mϕ into an inflammatory condition (GM-Mϕ or inflammatory macrophage).

While GM-Mϕ has condensed nuclei and relatively abundant cytoplasm rich in mitochondria, M-Mϕ has relatively smaller nuclei and less cytoplasm filled with lysosomes. Moreover, differential expression of Toll-like receptors (TLRs), C-type lectin receptors (CLRs), and cytosolic retinoid acid-inducible gene I (RIG-I)-like receptors (RLRs) have been observed [5]. While M-Mϕ is less sensitive to DV infection and do not produce interleukin-1beta (IL-1β) and IL-18, GM-Mϕ is highly susceptible to DV infection, release higher levels of tumor necrosis factor-alpha (TNF-α), and activate NLR family PYD-containing protein 3 (NLRP3) inflammasome to secrete IL-1β and IL-18 and become pyroptosis [5].

Inflammasome is composed of three components: the nucleotide-binding domain and leucine-rich repeat containing (NLR) proteins or the pyrin and HIN domain containing family member (PYHIN), apoptosis-associated speck-like protein containing a CARD (Asc), and pro-caspase-1. Activation of TLRs and CLRs results in the assembling of inflammasome to activate caspase-1, which further processes pro-IL-1β and IL-18 into mature forms and induces pyroptosis. Since inflammasomes play critical roles in Th17 activation and tissue damage during acute and chronic inflammation, GM-Mϕ may be an ideal *in vitro* model system to investigate the regulation of inflammasome activation by pathogens [6].

In addition to DV, the influenza virus elicits different responses from macrophage subsets, which is attributed to the distinct culture conditions *in vitro*. Cheung et al. demonstrated that H5N1 virus induced higher levels of TNF-α and interferon beta (IFNβ) than H1N1 and H3N2 in human macrophages differentiated by heat-inactivated autologous plasma [7]. However, Friesenhagen et al. suggested that induction of pro-inflammatory cytokines and type I IFNs were significantly abolished in H5N1-infected macrophages differentiated by cultivating monocytes in Teflon bags with RPMI-1640 medium, supplemented by 10% human AB serum than in H1N1-infected cells [8]. Thus, contradicting results seem come from distinct differentiation methods for macrophage subsets used in the study of host-pathogen interaction.

To address this important issue, cytokines optimal for macrophage differentiation is discussed and the current strategy of using human M-CSF to drive monocyte differentiation *in vitro* models to study host-pathogen interaction is re-visited.

### Signals for macrophage differentiation and activation

Macrophages can differentiate from either hematopoietic progenitor cells (HPC) or circulating monocytes, and display distinct phenotypes in host-pathogen interaction and the resolution of inflammatory reactions. Various cytokines and stimulatory signals are involved in the process. Monocytes differentiate into resting or inflammatory macrophages under the influence of M-CSF and GM-CSF, respectively [9], or into M1 and M2 by interferon-gamma (IFN-γ) and IL-4 priming, respectively [10-12]. Stimulation of macrophage subsets by pathogen-associated molecular patterns (PAMPs), damage-associated molecular patterns (DAMPs), or distinct resolution signal like IL-10, TGF-β and glucocorticoids, determine the consequence of host immune responses [12,13].

The M1 macrophages are responsible for the high levels of pro-inflammatory cytokines (i.e., TNF-α, IL-1β, and IL-6), IL-12 and IL-23, chemokines (Chemokine [C-C motif] ligand 5, CCL5, and C-X-C motif chemokine, CXCL10), and low levels of IL-10. As a result, M1 macrophages express strong anti-microbial activity and contribute to Th1 response. In contrast, M2 macrophages can be further classified into three major groups: M2a (induced by IL-4 or IL-13), M2b (induced by immune complexes and agonists of IL-1 receptors or TLRs), and M2c (induced by glucocorticoids or IL-10 or transforming growth factor beta, TGF-β). The M2 macrophages are characterized by low IL-12 and high IL-10 production. They are also responsible for resolving Th1 response and modulating tissue repair and remodeling [10,11,13].

### Role of M-CSF and GM-CSF in macrophage differentiation

The M-CSF controls the primary regulator of mononuclear phagocyte production *in vivo* and plays an essential role in the survival, proliferation, differentiation, and maturation of the macrophage myeloid lineage [14]. Mutation of M-CSF results in profound macrophage deficiency [15,16] similar to that observed in M-CSF receptor knock-out mice [17]. In contrast, disturbed hematopoiesis and deficient macrophages are not observed in GM-CSF knockout mice, even though GM-CSF-deficient mice develop abnormal lungs, including peri-brochovascular lymphocyte infiltration and surfactant accumulation in the alveoli. Moreover, opportunistic bacterial and fungal infections in lung tissue are the significant features of GM-CSF-deficient mice [18]. The GM-CSF^−/−^ mice is less able to control influenza virus infection than WT mice, and GM-CSF over-expression in lung epithelial cells in GM-CSF^−/−^ mice enhance mice survival after influenza virus infection [19]. This suggests that GM-CSF is necessary for host defense against pathogen invasion, while M-CSF is essential for driving monocyte differentiation into macrophage *in vivo*.

The M-CSF circulates at detectable levels in a steady state (<60 μg/animal) in normal healthy individuals. It is constitutively produced *in vitro* by several cell types, including fibroblasts, endothelial cells, stromal cells, macrophages, smooth muscle cells, and osteoblasts [20]. On the other hand, GM-CSF expression is spatially regulated and dramatically up-regulated at inflammation or infection sites [4], suggesting that macrophage differentiation during inflammatory reactions is under the influence of GM-CSF, which causes a massive increase in the macrophage population of the spleen and liver to induce hepato-splenomegaly [21]. Thus, the basal level of M-CSF is required to maintain the homeostasis of tissue macrophage through M-CSF signaling, while the local and temporal increase in GM-CSF, which inhibits M-CSF signaling during inflammation, polarizes monocytes to differentiate into inflammatory Mϕ during the inflammatory reaction and shift back to resting macrophages after the infection-induced inflammation is removed [4].

At present, inflammatory macrophages are considered to contribute to pathogen clearance by releasing many mediators like cytotoxic/pro-inflammatory/chemokine molecules, to eliminate pathogen infection and regulate other cell types while resting macrophages inhibit inflammation and initiate wound repair. Furthermore, excessive activation without resolution may result in tissue injury and even multisystem organ failure and death. The persistence of pro-inflammatory mediators may lead to the development of chronic inflammation. Therefore, the final outcome of the response of tissue injury or repair depends on the balance between two opposing forces affecting macrophages [4,13,22].

### Differential response of murine “inflammatory Mϕ” and “resting Mϕ” to lipopolysaccharide (LPS)

Recently, Fleetwood et al. compared the different responses of murine bone marrow-derived macrophages subsets GM-BMϕ and M-BMϕ to LPS stimulation. After LPS stimulation, GM-BMϕ preferentially produced TNF-α, IL-6, IL-12p70, and IL-23 whereas, while M-BMϕ generated more IL-10 and CCL2 under similar conditions. Interestingly, phenotypes of GM-BMϕ and M-BMϕ adopt the phenotype of other populations if pre-treated with M-CSF and GM-CSF, respectively. This indicates the plasticity of GM-BMϕ and M-BMϕ by M-CSF and GM-CSF, and further supports the argument that GM-BMϕ may be the dominant macrophage subset during the inflammatory reaction [23].

In addition to GM-CSF, type I IFN has crucial regulatory function in M-BMϕ and GM-BMϕ. Compared to GM-BMϕ, M-BMϕ constitutively express higher levels of IFN-β to enhance type I IFN signaling-dependent gene expression, including *Ccl5*, *Ccl12*, *Irf7*, *Stat1*, *Stat2* and *Cxcl10*. The autocrine type I IFN signaling in GM-BMϕ and M-BMϕ differentially regulates the production of M1 and M2 cytokines after LPS stimulation. These results indicate that endogenous and LPS-induced type I IFNs participate in regulating the phenotype and functions of M-BMϕ and GM-BMϕ [24].

Lacey et al. also revealed that IL-10^−/−^ M-BMϕ produced higher amounts of TNF, IL-6, IL-12p70, and IL-23p19 after LPS stimulation. Unlike M-BMϕ, GM-BMϕ has a similar response to LPS regardless of whether they are derived from wild type or IL-10^−/−^ mice [25]. Since the phenotype of M-BMϕ reflects steady-state macrophages, the selective influence of IFN-β and IL-10 is consistent with the concept that the micro-environment can influence the polarization of macrophage differentiation at the start of immune response, while GM-BMϕ in the inflammatory sites are resistant to the influence of exogenous cytokines such as IFN-β and IL-10. This partly explains the failure of IL-10 to suppress inflammatory reaction *in vivo*, where most activated macrophages behave as GM-BMϕ resistant to IL-10-mediated immuno-suppression in mouse models.

### Inflammasome activation and viral infections

Unlike other pro-inflammatory cytokines, the production of IL-1β and IL-18 is tightly controlled by the activation of inflammasome. External signals induce the assembling of inflammasome to activate caspase-1, which further processes pro-IL-1β and pro-IL-18 into mature cytokines and induce pyroptosis [6]. Three inflammasomes of the NLR family (NLRP1, NLR family CARD-containing protein [NLRC4], and NLRP3) and one PYHIN family member (absent in melanoma 2 [AIM2]) have been clearly identified to regulate IL-1β and IL-18 secretion in macrophages. The NLRP1 inflammasome senses anthrax lethal toxin while NLRC4 recognizes flagellin delivered through bacterial type III (T3SS) or type IV secretion systems (T4SS). The AIM2 inflammasome responds to cytosolic double-stranded DNA contributed by bacteria or virus. To date, the NLRP3 inflammasome is the well-characterized inflammasome that can sense many stimuli, including microbial stimuli (i.e., microbial lipopeptide, bacterial RNA, dsRNA) and particular molecules (e.g., amyloid deposit, silica, and aluminum salts) [26].

A recent study indicates that inflammasome activation plays critical roles in virus infections. Compared to the wild-type mice, IL-1RI^−/−^ mice have higher mortality after influenza virus infection [27]. IL-1β^−/−^ mice also express decreased immune response and increased viral load compared to wild-type mice after herpes simplex virus 1 (HSV-1) infection [28]. Like IL-1RI^−/−^ mice, IL-18^−/−^ mice have increased viral load and mortality after influenza virus infection compared to the wild-type mice [29]. In other study, administration of IL-18 before HSV-1 infection raises the survival rates of HSV-1-infected mice [30]. These indicate that IL-1β and IL-18 supports immune control against the influenza virus and protects against HSV-1-induced encephalitis.

To escape from host immunity, viruses also evolve distinct mechanisms to evade inflammasome activation, including 1) inhibiting inflammasome assembly (i.e., Kaposi's sarcoma-associated herpes virus and measles virus [MV]), 2) blocking caspase-1 function (e.g., orthopoxviruses and influenza virus), and 3) neutralizing IL-1β and IL-18 (e.g., vaccinia virus and cytoplasmic polyhedrosis virus) [31]. Thus, understanding of different activations of inflammasome in macrophage subsets may help illustrate the pathogenesis of dengue fever and dengue virus-induced lethal diseases.

### Distinct regulation of inflammasome activation by DV in human “inflammatory Mϕ” and “resting Mϕ”

Human M-Mϕ and dendritic cells (DCs) are the primary targets of DV infections [32-35]. Unlike DCs, which undergo apoptosis upon DV infection [35], human M-Mϕ survive for at least 45 days after DV infection, suggesting that M-Mϕ may be regarded as major sources of pro-inflammatory cytokines *in vivo*[32]. Chen et al. further demonstrated that DV activates M-Mϕ to secrete pro-inflammatory cytokines via CLEC5A, a DNAX-activating protein (DAP12)-associated C-type lectin, that is expressed on human M-Mϕ. Furthermore, antagonistic mAb against murine CLEC5A can prevent DV-induced pro-inflammatory cytokine release and lethal diseases *in vivo*[3]. This demonstrates that CLEC5A is crucial for the onset of DF and DHF/DSS, and M-Mϕ may be the most important cell subset in dengue infection.

However, whether or not human inflammatory macrophage subsets display distinct reactions to dengue virus infection has not been systemically addressed. In the study by Wu et al. [5], GM-Mϕ is more susceptible to DV infection than M-Mϕ (100-fold difference) and supernatant from DV-infected GM-Mϕ is more potent in increasing the permeability of endothelia cells, HMEC-1. While both cell types produce similar amounts of IFN-α, both IL-1β and IL-18 are undetectable in DV-infected M-Mϕ. In contrast, GM-Mϕ produces much higher amounts of TNF-α, IL-1β, and IL-18, and less IL-10. Furthermore, DV-infected GM-Mϕ can become pyroptosis due to caspase-1 activation. It is interesting to note that DV up-regulates NLRP3 expression without affecting NLRC4 and NLRP1, whereas NLRP3 siRNA inhibits DV-induced IL-1β and IL-18 secretion specifically in GM-Mϕ.

Since LPS-priming reportedly induce IL-1β transcription and enhance IL-1β production, Wu et al. further compared LPS-primed M-Mϕ and GM-Mϕ to DV infection. While LPS-priming dramatically increased production of IL-1β (25-fold), LPS-primed M-Mϕ still failed to produce detectable IL-1β and IL-18. Since IL-1β and IL-18 production is under the control of inflammasomes, this clearly shows the distinct regulation of inflammasomes in M-Mϕ and GM-Mϕ. Wu et al. further demonstrated that DV can trigger CLEC5A on GM-Mϕ to activate NLRP3 inflammasome, leading to the secretion of IL-1β and IL-18 [5]. This observation further indicates CLEC5A may play a critical role in DV-induced inflammasome activation.

**Figure 1:**
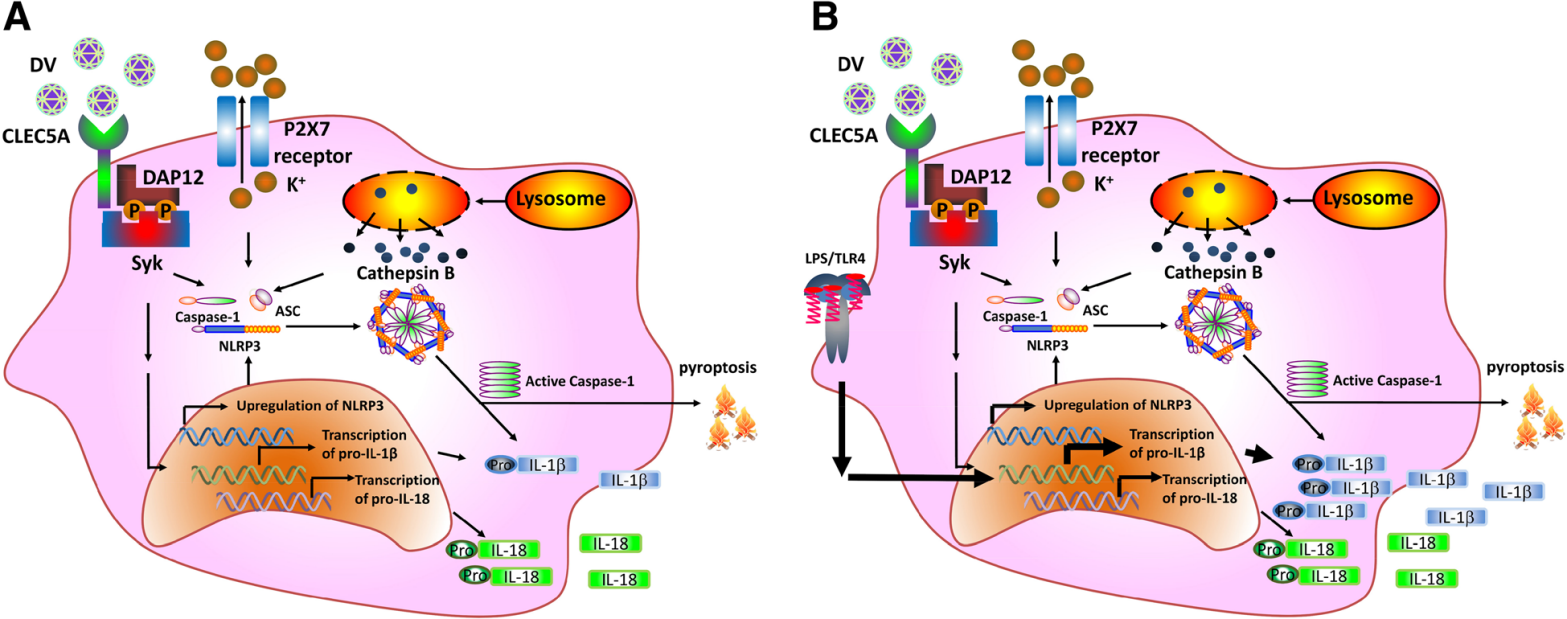
**Activation of NLRP3 inflammasome in DV-infected GM-Mϕ.** GM-Mϕ is infected with DV directly **(A)**, or after LPS priming **(B)**. DV binding to CLEC5A recruits DAP12, which is phosphorylated by Src, and then activates Syk. Activated Syk induces the transcription of IL-1β, IL-18, and NLRP3 to activate inflammasome and caspase-1, leading to cell death (pyroptosis) and cleavage of pro-IL-1β and pro-IL-18. Secondary signaling, potassium efflux, and lysosome cathepsin B are also involved in NLRP3 inflammasome activation and the release of IL-1β and-IL-18 from DV–infected GM-Mϕ. LPS priming further enhances the transcription of IL-1β (significantly), IL-18 (slightly), and NLRP3 (slightly), and further increases the secretion of IL-1β. DV, dengue virus; NLRP, NLR family PYD-containing protein; LPS, lipopolysaccharides; IL-1β, interleukin -1beta.

**Figure 2:**
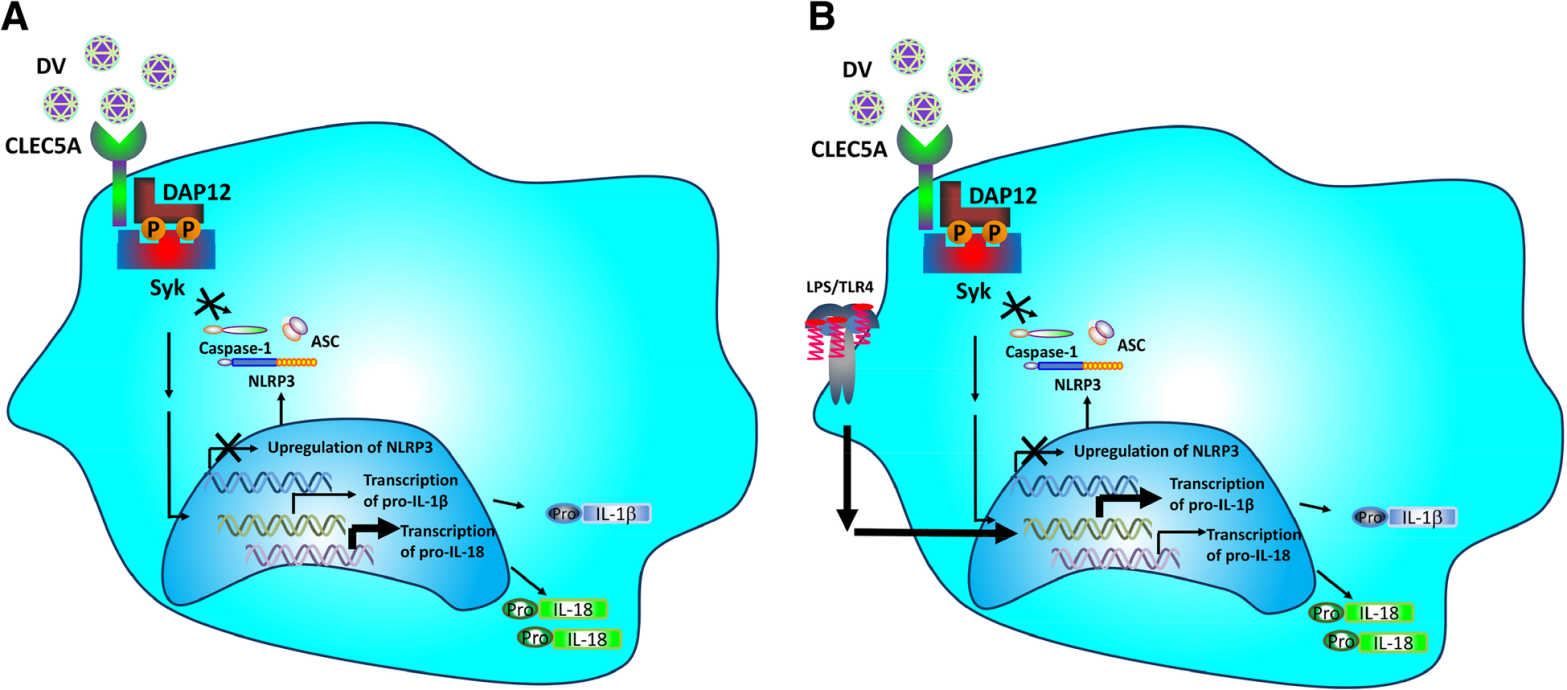
**Activation of NLRP3 inflammasome in DV–infected M-Mϕ.** M-Mϕ is incubated with DV directly **(A)**, or after LPS priming **(B)**. DV activates Syk via CLEC5A to up-regulate the transcription of IL-1β (slightly) and IL-18 (significantly), but is unable to induce NLRP3 transcription. LPS priming further up-regulates the transcription of IL-1β, but down-regulates IL-18 transcription. LPS priming cannot enhance the transcription of NLRP3. NLRP3 inflammasome is not activated in DV-infected M-Mϕ, and thus, is unable to activate caspase-1 to process pro- IL-1β and pro-IL-18. NLRP, NLR family PYD-containing protein; DV, dengue virus; LPS, lipopolysaccharides; IL-1β, interleukin -1beta.

Supernatants from DV-infected GM-Mϕ are more potent than that from DV-infected M-Mϕ in increasing the permeability change of endothelial cells. As such, GM-Mϕ seems more critical than M-Mϕ in the pathogenesis of dengue fever, dengue hemorrhagic fever, and dengue shock syndrome. The DV-induced inflammasome activation pathways in GM-Mϕ and M-Mϕ are summarized in Figures 1 and 2[5].

In addition to different responses of human M-Mϕ and GM-Mϕ to DV, Verreck et al. [36] cultured human GM-Mϕ and M-Mϕ to study their distinct roles in mycobacteria. They found that GM-Mϕ secreted high levels of IL-23 (p40/p19) but not IL-12 (p40/p35) after mycobacterial infection, while a secondary signal, IFN-γ, induced IL-12p35 transcription and IL-12 production. In contrast to GM-Mϕ, M-Mϕ predominantly produced IL-10, but not IL-12 and IL-23. Also, only GM-Mϕ, not M-Mϕ, supported Th1 response after mycobacterial infection. These results indicate that IL-23, but not IL-12, is the major type 1 cytokine produced by mycobacteria-stimulated GM-Mϕ, and that GM-Mϕ and M-Mϕ also play essential roles in anti-mycobacterial immunity.

### Differential expression of innate immunity receptors and inflammasomes in human macrophage subsets

**Figure 3:**
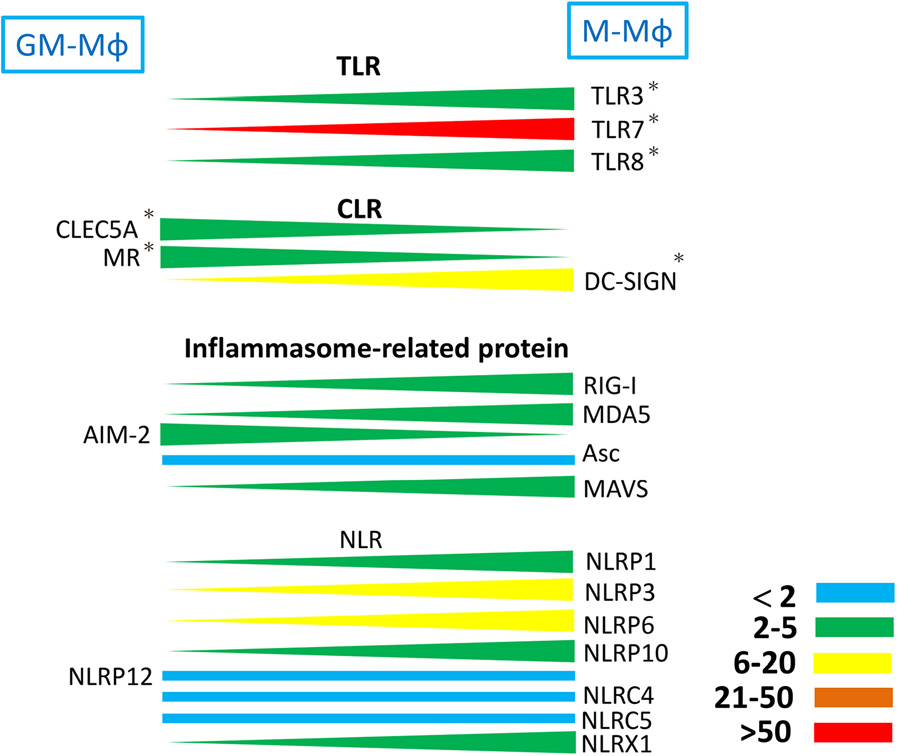
**Relative expression levels of TLRs, CLRs, and inflammasome components between GM-Mϕ and M-Mϕ.** Expression levels of these genes are determined by real time PCR and the differential expression levels between GM-Mϕ and M-Mϕ are indicated in color: blue (<2 fold), green (2–5 fold), blue (6–20 fold), brown (21–50 fold), and red (>50 fold). *Indicates the protein expression level for the indicated receptor as confirmed by flow cytometry. TLR, Toll-like receptor; CLR, C-type lectin receptor.

Recently, a comprehensive study was conducted to shed light on the expression of TLRs, CLRs, and inflammasome components involved in recognizing DV in human M-Mϕ and GM-Mϕ. Before incubation with DV, baseline levels of TLRs (TLR 3, 7, and 8), Dendritic Cell-Specific Intercellular adhesion molecule-3-Grabbing Non-integrin (DC-SIGN), and most of the inflammasomes receptors (except AIM-2 and NLRP12) were higher in M-Mϕ. Higher expression levels of CLEC5A and MR were found in GM-Mϕ (Figure 3).

**Figure 4:**
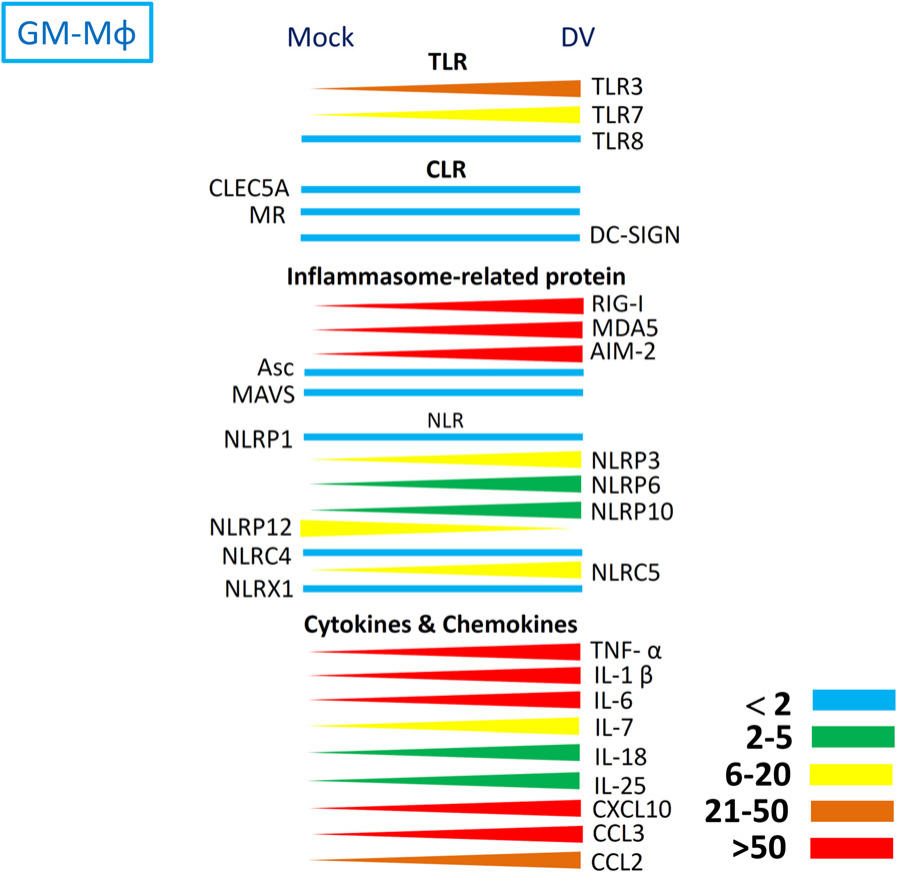
**Expression levels of TLRs, CLRs, and inflammasome components in GM-Mϕ after DV infection.** After incubation with DV for 24 hours, the expression levels of each gene were determined by real-time PCR. The difference in expression levels between mock and DV is indicated in color: blue (<2 fold), green (2–5 fold), blue (6–20 fold), brown (21–50 fold), and red (>50 fold). TLR, Toll-like receptor; CLR, C-type lectin receptor; DV, dengue virus.

**Figure 5:**
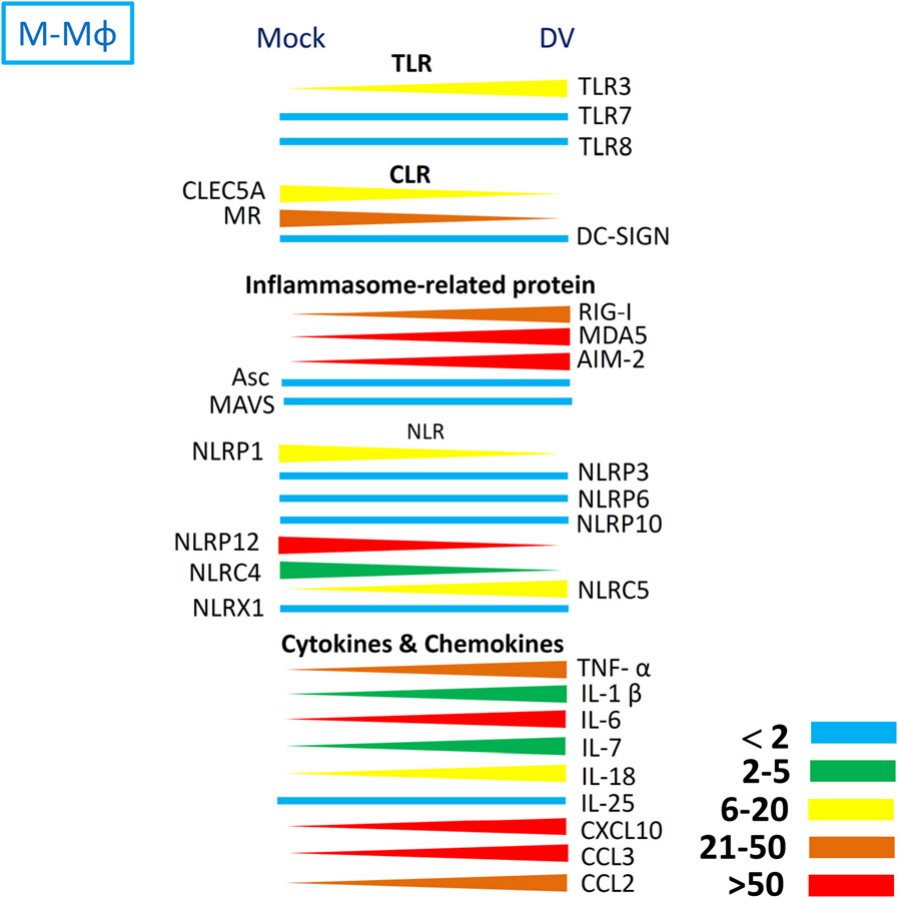
**Expression levels of TLRs, CLRs, and inflammasome components in M-Mϕ after DV infection.** After incubation with DV for 24 hours, the expression levels of each gene were determined by real-time PCR. The difference in expression levels between mock and DV are indicated in color: blue (<2 fold), green (2–5 fold), blue (6–20 fold), brown (21–50 fold), and red (>50 fold). TLR, Toll-like receptor; CLR, C-type lectin receptor; DV, dengue virus.

Infection with DV up-regulated the expression levels of pro-inflammatory cytokines, chemokines, TLRs, and most members of NLRs. In contrast, the expressions of CLEC5A, MR, ASC, mitochondrial antiviral signaling protein (MAVS), and members of NLRs (including NLRP1, NLRP12, NLRC4, and the NLR family member X1 [NLRX1]) were down-regulated in both M-Mϕ and GM-Mϕ (Figures 4 and 5).

**Figure 6:**
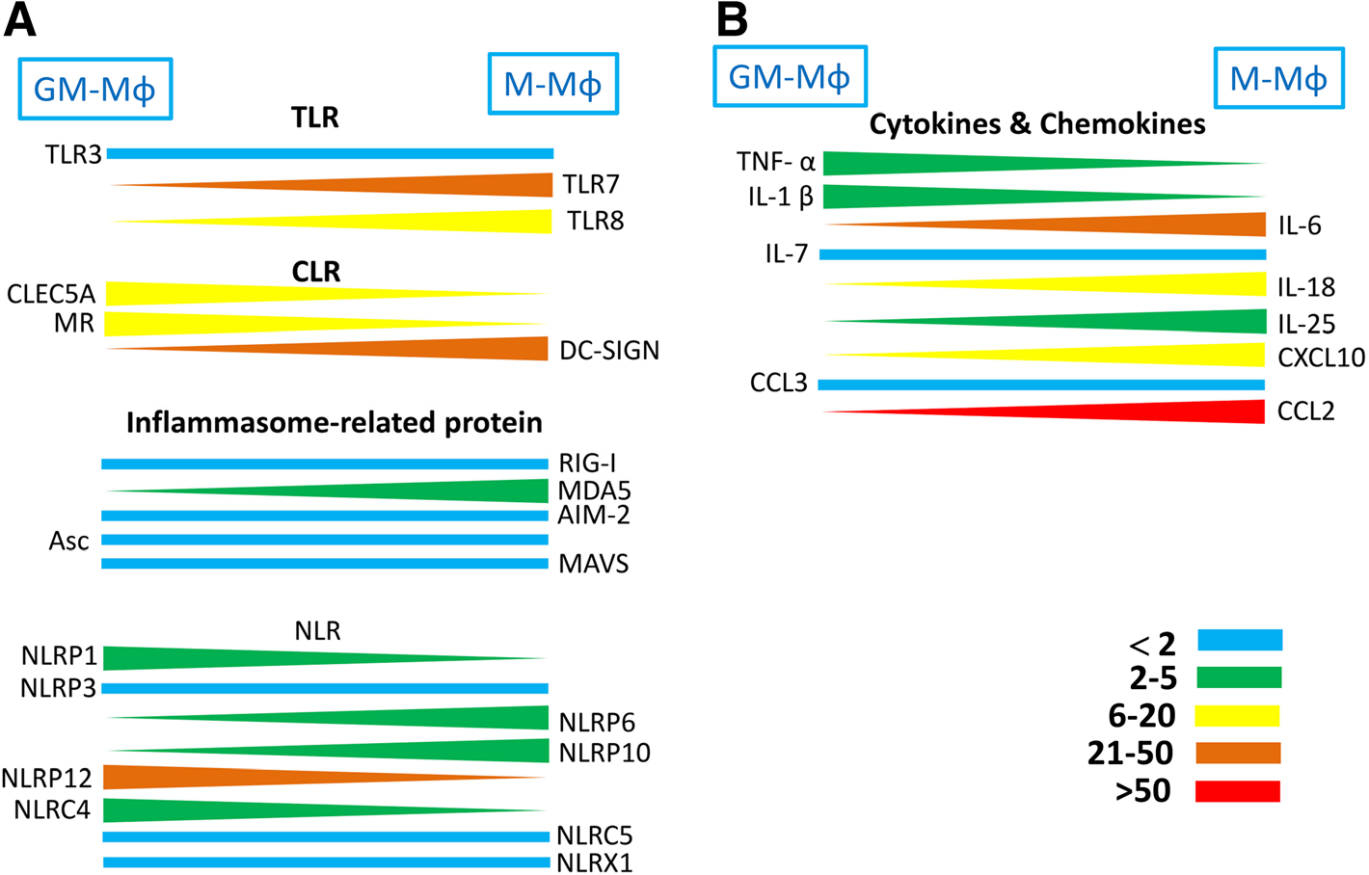
**Relative expression levels of TLRs, CLRs, inflammasome components, cytokines, and chemokines between GM-Mϕ and M-Mϕ after DV infection.** The difference in gene expression levels between GM-Mϕ and M-Mϕ are indicated in color: blue (<2 fold), green (2–5 fold), blue (6–20 fold), brown (20–50 fold), and red (>50 fold). TLR, Toll-like receptor; CLR, C-type lectin receptor; DV, dengue virus.

Although the expression profiling of CLRs, TLRs, inflammasomes, pro-inflammatory cytokines, and chemokines modulated by DV is similar, the relative expression levels of genes between M-Mϕ and GM-Mϕ are enormous after DV infection (Figure 6). The expressions of CLEC5A, MR, NLRP1, and NLRC4 in GM-Mϕ are also higher (2- to 20-fold) than in M-Mϕ. It is surprising that NLRP12 expression is much higher (30-fold) in GM-Mϕ than in M-Mϕ. In addition, although NLRP3 gene expression in DV-infected GM-Mϕ is slightly higher than in M-Mϕ (Figure 6), DV infection induces NLRP3 gene up-regulation (Figure 4). This effect is not observed in M-Mϕ (Figure 5).

In contrast, expressions of TLR8, melanoma differentiation-associated antigen 5 (MDA5), NLPRP6, NLRP10, IL-18, IL-25, and CXCL10 (Interferon gamma-induced protein 10, IP-10) is higher (2- to 20-fold) in M-Mϕ than in GM-Mϕ. It is interesting to note that expressions of TLR7, DC-SIGN, IL-6, and CCL2 (monocyte chemotactic protein-1, MCP-1) are up-regulated by more than 30-fold in DV-infected M-Mϕ (Figure 6). The IL-18 mRNA expression is higher in DV-infected M-Mϕ, but IL-18 is still not detectable in DV-infected M-Mϕ supernatant. This suggests the presence of a negative regulator controlling NLRP3 activation in DV-infected M-Mϕ. The different expression profiling of cytokines and innate immunity receptors/sensors between M-Mϕ and GM-Mϕ further supports the notion that these two subsets have distinct functions in DV infection.

**Figure 7:**
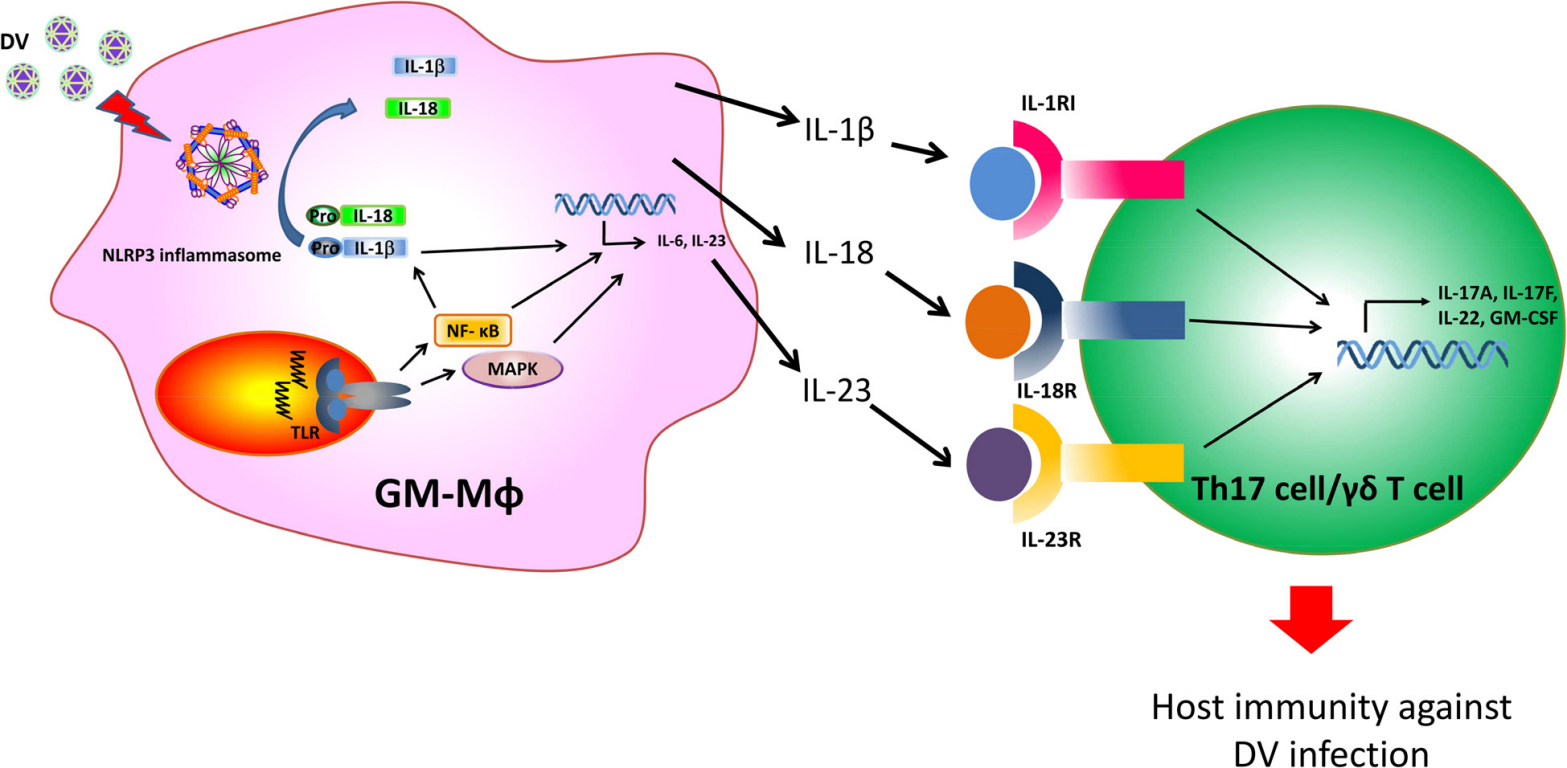
**The combination of NLRP3 inflammasome-processed cytokines and IL-23 during DV infection induced the production of IL-17 from Th17/γδ Tcells.** Stimulation of TLRs in GM-Mϕ with DV can induce the activation of NF-κB and MAPK, which promotes the transcription of a range of pro-inflammatory cytokines. NLRP3 inflammasome-activated caspase-1 further processes the pro-IL-1β and pro-IL-18 into their mature cytokine form, IL-1β and IL-18. IL-1β can also enhance the production of IL-23 and IL-6. The released IL-1β, IL-18, and IL-23 induce Th17/γδ T cells to produce pro-inflammatory cytokines which are responsible for host immune responses against DV infection.

Aside from mediating immune response to pathogen infection, IL-1β and IL-18 play an important role in driving adaptive immunity during infection. The collaboration of IL-1β, IL-18, and IL-23 triggers the secretion of IL-17 from Th17 cells and IL-17-secreting γδ T cells. As a result, regulation for the synthesis and production of IL-1β and IL-18 is the key point for modulating IL-17-associated diseases. Recent studies have shown that IL-1β can induce the expression of IL-23 and the secretion of IL-6, which is essential for Th17 cells differentiation [37-39]. The potential role of IL-1β and IL-18 released from DV-infected GM-Mϕ in the Th17 differentiation is shown in Figure 7.

## Conclusions

IL-1β is the most potent endogenous pyrogen [40,41] and is essential for the differentiation of Th17 and other cell subsets to fight pathogens [42]. IL-18 serum level correlates with thrombocytopenia and dengue hemorrhage [43]. Recent studies further demonstrates that serum IL-1β and IL-18 levels correlate with susceptibility to dengue [44,45]. Thus, GM-Mϕ seems to be crucial in understanding the pathogenesis of DV-induced lethal diseases. In our recent study [5], we observed the differential responses of M-Mϕ and GM-Mϕ to DV, such as infection rate, and the potential ability for IL-1β and IL-18 production. The differential expression level of MR may determine the differential infection rate because of its strong binding to DV, while the distinct regulation of inflammasome activation in M-Mϕ and GM-Mϕ contribute to the differential production of IL-1β and IL-18. In GM-Mϕ, activation of Syk-coupled CLEC5A induces the transcription of pro-IL-1β and NLRP3 as well as the activation of caspase-1 during DV infection. Moreover, LPS priming further enhances IL-1β production by increasing pro-IL-1β transcription and translation (Figure 1). In contrast, transcription of pro-IL-1β, NLRP3 and caspase-1 activation are not observed in M-Mϕ, thus fails to produce mature IL-1β/IL-18 even with LPS priming (Figure 2).

In contrast, avian influenza virus (HPAIV)-infected macrophages can escape inflammasome activation and IL-1β production because of the lack of viral M2 protein required for NLRP3 inflammasome activation in other influenza virus stains [8,46]. This escape mechanism for HPAIV may affect the immune response of human macrophages and enhance the possibility for HPAIV causing systemic infection and a cytokine storm in the later stage of infection.

Aside from DV and the influenza virus, intracellular bacteria (like *Mycobacterium, Salmonella*, and *Listeria monocytogenes*) and fungi (such as *Candida albicans* and *Aspergillus fumigatus*) also invade and replicate in macrophages [26]. However, most studies incubate pathogens with M-Mϕ *in vitro* and do not compare the different responses of GM-Mϕ and M-Mϕ. Whether the phenomenon observed *in vitro* reflects event *in vivo* need to be re-evaluated.

Inflammasome activation is crucial for starting innate immunity and controlling host immune response to PAMPs and DAMPs [47]. GM-Mϕ is invaluable for the identification of novel genes involved in regulating inflammasome activation. By comparing the gene expression profiling in GM-Mϕ and M-Mϕ using microarray, it becomes possible to find positive and negative regulators to control inflammasome activation and inhibition, and help identify novel therapeutic targets for treating human diseases due to exaggerated activation or inhibition in the future.

## Abbreviations

M-CSF: Macrophage colony-stimulating factor; GM-CSF: Granulocyte macrophage colony-stimulating factor; DF: Dengue fever; DHF: Dengue hemorrhagic fever; DSS: Dengue shock syndrome; HSCs: Hematopoietic stem cells; TLR: Toll-like receptor; RLR: Retinoid acid-inducible gene I (RIG-I)-like receptors; CLR: C-type lectin receptor; MR: Mannose receptor; DC-SIGN: Dendritic Cell-Specific Intercellular adhesion molecule-3-Grabbing Non-integrin; RIG-I: Retinoic acid inducible gene-I; MDA5: Melanoma differentiation-associated antigen 5; AIM2: Absent in melanoma 2; MAVS: Mitochondrial anti-viral signaling protein; ASC: Apoptosis-associated speck-like protein containing a CARD; NLR: Nucleotide-binding domain leucine-rich repeat; PYHIN: Pyrin and HIN domain containing; NLRP: NLR family PYD-containing protein; NLRC: NLR family CARD-containing protein; NLRX1: NLR family member X1; DAP12: DNAX-activating protein 12; TNF-α: Tumor necrosis factor; IL-1β: Interleukin-1 beta; IFNβ: Interferon beta; IFN-γ: Interferon-gamma; PAMPs: Pathogen-associated molecular patterns; DAMPs: Damage-associated molecular patterns; Th1: T-helper type 1; LPS: Lipopolysaccharides; CCL5: (Chemokine [C-C motif] ligand 5; CXCL10: C-X-C motif chemokine 10; TGF-β: Transforming growth factor beta; IP-10: Interferon gamma-induced protein 10; MCP-1: Monocyte chemotactic protein-1; NF-κB: Nuclear factor-κB; HSV-1: Herpes simplex virus 1; KSHV: Kaposi's sarcoma-associated herpes virus; MV: Measles virus; CPV: Cytoplasmic polyhedrosis virus.

## Competing interests

The authors declare that they have no competing interests.

## Authors’ contributions

MFW and STC were involved in literature review, discussion, and drafting of the manuscript. SLH initiated the concept and wrote the draft of this manuscript. All of the authors read and approved the final manuscript.
